# Growth-factor reduced Matrigel source influences stem cell derived brain microvascular endothelial cell barrier properties

**DOI:** 10.1186/s12987-016-0030-5

**Published:** 2016-04-12

**Authors:** Ronak Patel, Abraham J. Alahmad

**Affiliations:** Department of Pharmaceutical Sciences, School of Pharmacy, Texas Tech University Health Sciences Center, 1300 South Coulter Street, Amarillo, TX 79106 USA

**Keywords:** Stem cells, Blood–brain barrier, Matrigel, Barrier function, Drug transporters

## Abstract

**Background:**

Patient-derived induced pluripotent stem cells (iPSCs) are an innovative source as an in vitro model for neurological diseases. Recent studies have demonstrated the differentiation of brain microvascular endothelial cells (BMECs) from various stem cell sources, including iPSC lines. However, the impact of the culturing conditions used to maintain such stem cell pluripotency on their ability to differentiate into BMECs remains undocumented. In this study, we investigated the effect of different sources of Matrigel and stem cell maintenance medium on BMEC differentiation efficiency.

**Methods:**

The IMR90-c4 iPSC line was maintained on mTeSR1 or in essential-8 (E-8) medium on growth factor-reduced (GFR) Matrigel from three different manufacturers. Cells were differentiated into BMECs following published protocols. The phenotype of BMEC monolayers was assessed by immunocytochemistry. Barrier function was assessed by transendothelial electrical resistance (TEER) and permeability to sodium fluorescein, whereas the presence of drug efflux pumps was assessed by uptake assay using fluorescent substrates.

**Results:**

Stem cell maintenance medium had little effect on the yield and barrier phenotype of IMR90-derived BMECs. The source of GFR-Matrigel used for the differentiation process significantly impacted the ability of IMR90-derived BMECs to form tight monolayers, as measured by TEER and fluorescein permeability. However, the Matrigel source had minimal effect on BMEC phenotype and drug efflux pump activity.

**Conclusion:**

This study supports the ability to differentiate BMECs from iPSCs grown in mTeSR1 or E-8 medium and also suggests that the origin of GFR-Matrigel has a marked inpact on BMEC barrier properties.

## Background

The blood–brain barrier (BBB), or neurovascular unit, provides a stable and defined microenvironment for the central nervous system (CNS). Among the different types of cells forming the BBB, brain microvascular endothelial cells (BMECs) provide both a physical and chemical barrier, tightly regulating the diffusion of water, ions and lipophilic compounds into the CNS [1–4].

In vitro models of the BBB are valuable tools for drug discovery research as they provide an insight into the diffusion profile of existing and prospective drug candidates targeting the CNS [2, 5]. However, the current use of in vitro models of the human BBB based on primary cultures and immortalized cell lines remains hampered by their limited barrier tightness [6].

Recently, several studies have documented success in differentiating BMECs from stem cell sources, including human pluripotent stem cells such as embryonic stem cells (hESCs), hematopoietic stem cells (HSCs) and induced pluripotent stem cells (iPSCs) [7–10]. Such stem cell-derived BMECs share similarities with established models including the expression of tight junction proteins, the formation of a tight monolayer and the expression of functional drug transporters. Several parameters have been described as important for the generation of human pluripotent stem cell-derived BMECs in order to achieve barrier tightness suitable for drug permeability screening. These include cell density [11], the addition of retinoic acid [7], and co-culture with astrocytes and neurons [7, 8]. However, the ability to translate the differentiation protocol for patient-derived iPSCs may be hampered by variations in stem cell maintenance protocols. For instance, human pluripotent stem cells (hPSCs) grown and maintained in a feeder-free system are routinely grown on growth factor reduced (GFR) Matrigel- or vitronectin-N (VTN)-coated tissue culture plastic surfaces [12, 13], supplemented with mTeSR1 or essential 8 medium (E8) [12, 13]. GFR Matrigel constitutes a complex extracellular matrix (ECM) mixture secreted by Engelbreth–Holm–Swarm mouse sarcoma cells with an approximate composition of 60 % laminin, 30 % collagen IV and 8 % entactin as well as other undefined ECM components [14]. Because of its complexity, Matrigel remains undefined and may constitute a source of variability that can impact hPSC-derived BMEC differentiation efficacy [15]. However, the effect of other culturing conditions (ECMs and media) on the outcome of hPSC-derived BMEC differentiation remains undocumented. In this study, we investigated the effects of different ECMs and stem cell maintenance medium on IMR90-c4 human iPSC line, a cell line with a highly-documented BMEC differentiation profile.

## Methods

The undifferentiated IMR90 (IMR90-c4) iPSC line [13] (WiCell, Madison, WI, USA) was maintained in mTeSR (mTeSR1, Stem Cell Technologies, Vancouver BC, Canada) or in essential-8 medium (E-8, ThermoFisher, Waltham, MA, USA) on hESC-qualified GFR-Matrigel (C-Matrigel, Corning, Corning, NY, USA) [13].

Undifferentiated IMR90-c4 iPSC colonies were routinely maintained on 6-well tissue culture plates (Corning) coated with C-Matrigel in presence of mTeSR1 or E-8 medium. Prior to differentiation, cells were dissociated as single cells using Accutase cell detachment solution (Corning) and seeded at a seeding density of 8 × 10^3^ cells/cm^2^ (B-Matrigel) or 20 × 10^3^ cells/cm^2^ on tissue culture plates (Corning). Plates were coated with one of the following hESC-qualified GFR-Matrigels at a concentration 8.3 µg/cm^2^ for B-Matrigel, (BD Biosciences, Fairleigh, NJ, USA) and T-Matrigel, (Trevigen, Gaithersburg, MD, USA) or at a dilution indicated by the manufacturer for C-Matrigel; L-Matrigel, and Vitronectin (ThermoFisher, Waltham, MA, USA). This study was conducted with at least two distinct ECM batches serial numbers. After 5 days of culture, IMR90 iPSCs became differentiated into BMECs as previously reported [7, 11]. In brief, cells were maintained in unconditioned medium (UM; DMEM/F12 with 15 mM HEPES supplemented with 20 % KO serum replacement, 1 % MEM non-essential aminoacids, and 0.5 % Glutamax I, ThermoFisher) and 0.1 mM β-mercaptoethanol (Sigma-Aldrich, St Louis, MO, USA) for six consecutive days, with the medium replaced daily. After 6 days of differentiation, IMR90-derived BMECs were further matured in EC differentiation medium (EC serum free medium (ThermoFisher), supplemented with 1 % platelet-poor derived plasma serum (ThermoFisher), 20 µg/mL human basic fibroblast growth factor (R&D Systems) and 10 µM all-trans retinoic acid (Sigma-Aldrich) for 2 days. After 8 days of differentiation, cells were enzymatically dissociated and seeded at a density of 10^6^ cells/cm^2^ on 12-well transwell polyester cell culture inserts (0.4 µm pore size) coated with collagen from human placenta (Sigma-Aldrich) and fibronectin from bovine plasma (Sigma-Aldrich) at concentrations of 80 and 20 µg/cm^2^ respectively as previously published [7, 11]. After 24 h (day 9), BMECs were maintained in EC differentiation medium containing only 1 % platelet-poor plasma-derived serum. On day 10, 48 h after seeding, transendothelial electrical resistance (TEER) was assessed using an EVOM STX2 chopstick electrode (World Precision Instruments, Sarasota, FL, USA) and by measuring permeability (Pe) to sodium fluorescein solution (1 μg/mL, Sigma-Aldrich) using the clearance slope method as previously reported [7, 11, 16].

The immunostaining procedure on fixed cells was identical to that previously published [7, 11]. Cells were visualized on an Olympus IX81 inverted epifluorescence microscope. Photomicrographs were acquired using Slidebook 4.5 (Intelligent Imaging Innovation, Denver, CO, USA) and processed with ImageJ (NIH, Bethesda, MD, USA).

Drug efflux pump activity was assessed using the same accumulation assays as previously reported [7, 11]. In brief, BMECs monolayers were incubated in presence of 10 µM Rhodamine 123 and 10 µM BODIPY-FL prazosin (Sigma-Aldrich), 5 µM doxorubicin or 10 µM CM-DCFDA for 1 h at 37 °C. For experiments involving inhibitors, cells were pre-incubated in presence of cyclosporine A (CsA), 5 µM, Ko143, 1 µM, or MK571, 10 µM (Sigma-Aldrich) for 1 h prior to the experiment and maintained during the whole procedure. Cells were washed with ice-cold PBS and lysed using RIPA extraction buffer (ThermoFisher). The fluorescence of the cell extract was assessed using a fluorescent plate reader (SynergyMX2, BioTek, Burlington, VT, USA). Raw fluorescence units (RFU) were adjusted to the cell extract protein concentration (as determined by BCA assay) to determine the drug uptake (expressed as RFU/µg protein). The drug uptake in untreated (without inhibitors) monolayers was arbitrarily set at 100 % and identified as control. The drug uptake in treated monolayers (with pharmacological inhibitors) was normalized to their respective controls, as previously published [7, 11].

Data are expressed as mean ± SD from a minimum of six experiments (two distinct ECM batches, three distinct differentiation passages for each batch. For each passage, experiments were done with two technical duplicates). Statistical analysis was performed using one-way ANOVA using Prism 6.0 statistical package (Graphpad Software, La Jolla, CA, USA).

## Results

### Experiments using mTeSR medium

**Fig. 1:**
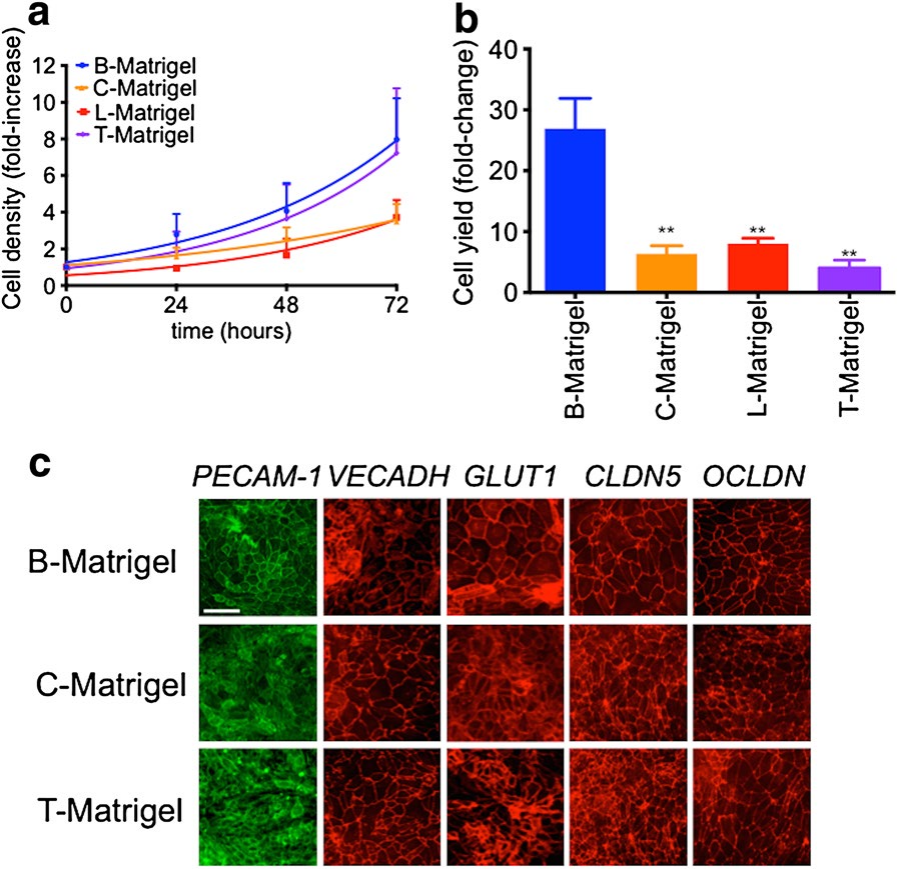
Effects of Matrigel origin on IMR90 iPS cell growth and BMEC differentiation using mTeSR medium. **a** Undifferentiated IMR90 cells maintained in mTeSR were dissociated as single cells seeded on Matrigel from BD Biosciences (B-Matrigel, *blue*), from Corning (C-Matrigel, *orange*), from ThermoFisher (L-Matrigel, *red*) or from Trevigen (T-Matrigel, *purple*) at 20 × 10^3^ cells/cm^2^. After 24, 48 and 72 h following cell seeding, IMR90 iPSC colonies were dissociated and counted using a cell hemocytometer and Trypan blue exclusion assay. **b** BMEC yield after 8 days of differentiation obtained by calculating the ratio of cell density at 8 days of differentiation versus the initial cell density seeded at day 0, ** denotes *P* < 0.01 in comparison to B-Matrigel. **c** Representative immunostained micrographs of PECAM-1, VE-cadherin, Glut-1 Claudin 5 and Occludin expression in purified BMEC monolayers 48 h post-purification. *Note* the formation of a monolayer as marked by defined tight junction complexes in one focal plane and the absence of junctional cellular overlap. *Scale bar* = 20 µm

We first investigated the impact of different Matrigel sources on undifferentiated IMR90 stem cell growth and maintenance in mTeSR medium at a seeding density of 20 × 10^3^ cells/cm^2^ (Fig. 1a). In our hands, IMR90 iPSC colonies grown on B-Matrigel, L-Matrigel  and T-Matrigel showed similar doubling times (27.28, 26.69 and 24.49 h respectively), however IMR90 cells grown on C-Matrigel showed a much slower growth rate, with an average doubling time of 42.30 h. We found that culturing IMR90 iPSCs on C-Matrigel and T-Matrigel with a starting seeding cell density of 8 × 10^3^ cells/cm^2^ as previously reported [11], failed to achieve a complete differentiation and we noted a significant cell loss after 3–4 days of growth in unconditioned medium (UM, data not shown). A satisfactory differentiation was only achieved when we set our seeding cell density at 20 × 10^3^ cells/cm^2^ and allowed our colonies to grow for 5 days. At this seeding density, the yield (calculated as the number of cells harvested at day 8 of differentiation versus the initial seeding density at day 0) was approximately 26.92 ± 4.69-fold increase in B-Matrigel group, but was lower in C- and T-Matrigel, with increases of 6.30 ± 1.32 and 4.03 ± 1.32-fold, respectively (Fig. 1b). Upon purification, we noted a homogenous BMEC cell monolayer in all three groups, as these cells expressed markers representative of the vascular endothelial lineage (PECAM-1, VE-cadherin), and also the expression of BBB markers (GLUT1, claudin-5, occludin), consistent with the existing literature (Fig. 1c) [7, 8, 11]. Taken together, our data suggest that Matrigel from different sources may affect the outcome in terms of BMEC differentiation and yield.

**Fig. 2:**
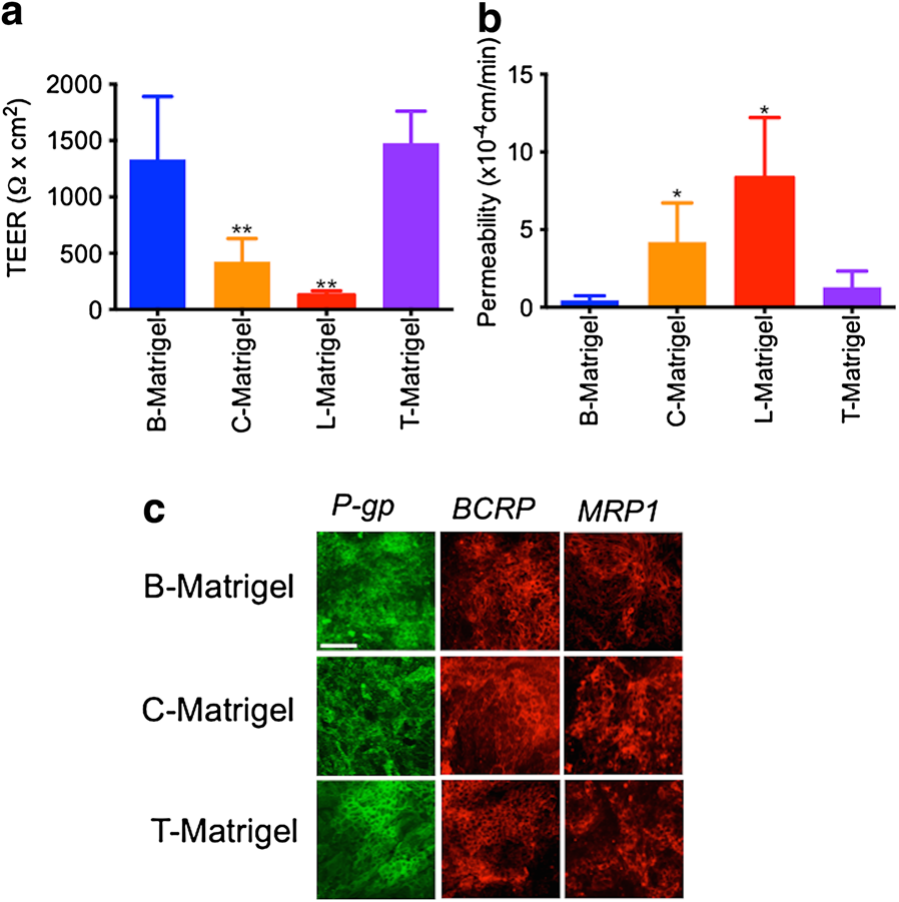
Matrigel origin impacts BMECs barrier properties. **a**, **b** TEER and sodium fluorescein permeabilities in IMR90-derived BMECs differentiated on B-, C- or T-Matrigel. *Note* the degraded barrier function of cells grown on C-Matrigel compared to the two other groups. * and ** denotes *P* < 0.05 and *P* < 0.01 in comparison to B-Matrigel. **c** Representative immunostained micrographs of P-gp, BCRP and MRP1 expression in purified BMECs monolayers. *Scale bar* = 40 µm

Next, we assessed the barrier properties of such monolayers in monocultures by measuring both transendothelial electrical resistance (TEER) and paracellular permeability to sodium fluorescein (NaF). On B-Matrigel, IMR90 showed TEER (Fig. 2a) values of 1332 ± 558 Ω cm^2^. In contrast, BMECs differentiated on C-Matrigel displayed a much lower TEER and the average electrical resistance measured was 424.3 ± 205.1 Ω cm^2^. Finally, on T-Matrigel we noted showed TEER values of 1439 ± 281.4 Ω cm^2^. Using a different technique to confirm such differences in the barrier function, we measured changes in paracellular permeability using sodium fluorescein (NaF) as a paracellular marker (Fig. 2b). In our hands, we noted permeability (Pe) values of 0.44 × 10^−4^ cm/min on monolayers differentiated on B-Matrigel. Cells differentiated on C-Matrigel showed a tenfold higher permeability, with an average Pe value of 4.20 × 10^−4^ cm/min, whereas cells grown on T-Matrigel had a Pe value of 1.57 × 10^−4^ cm/min.

**Fig. 3:**
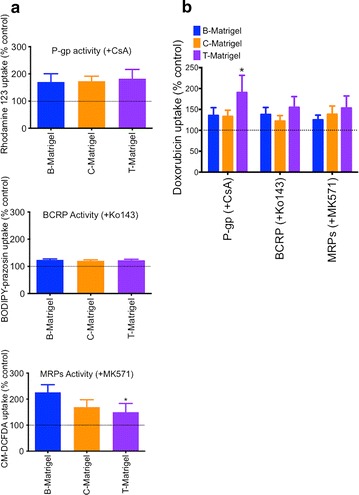
Matrigel origin has a minor impact on BMECs drug efflux activity. **a** Drug uptake of rhodamine 123, BODIPY FL-prazosin and CM-DCFDA in presence of 5 μM cyclosporine A (CsA, P-gp inhibitor), 1 μM Ko143 (BCRP inhibitor) or 10 μM MK571 (pan-MRP inhibitor), respectively. Fluorescence values in controls (no inhibitor) were arbitrarily set to 100 %, * and ** denote *P* < 0.05 and *P* < 0.01 in comparison to B-Matrigel. **b** Doxorubicin accumulation profile in presence of CsA, Ko143 or MK571. Doxorubicin net uptake was calculated as previously described

In addition, we investigated the expression (Fig. 2c) and activity (Fig. 3) of P-glycoprotein (P-gp), breast cancer resistant protein (BCRP) and multidrug resistant protein (MRP) by immunocytochemistry and by drug accumulation assay, respectively. In our hands, all groups had positive expression for P-gp, BCRP and MRP1 (Fig. 2c); all three proteins were detected in our monolayers and this was consistent with the existing literature [7, 8, 11]. We did not notice any dramatic differences in immunoreactivity between the three groups. Thus we consider there are similar protein expression levels in all groups. To demonstrate that Matrigel had little effect on drug efflux pump activity, we assessed changes in efflux activity by measuring, in the presence or absence of inhibitors, uptake of rhodamine 123, FL-BODIPY prazosin, and CM-DCFDA as these fluorescent dyes are known substrates for P-gp [17], BCRP [18–20] and MRPs [21], respectively (Fig. 3). The net uptake (the difference in fluorescence between untreated group designated 100 %, and inhibitor-treated group), was not significantly different for P-gp-mediated efflux between the groups (Fig. 3a, upper panel). However, we noted a 1.7 fold increase in uptake of rhodamine 123 following inhibition with 5 µM CsA: this increase was consistent with the previous literature [7, 8, 11]. A similar outcome was observed in the net uptake of FL-BODIPY prazosin following inhibition with Ko143 (Fig. 3a, middle panel). However, we noted a slight but significant difference in net uptake of CM-DCFDA in that cells grown on B-Matrigel showed a 2.3 fold-increase in uptake in presence of MK571 compared to control, whereas cells grown on T-Matrigel showed only a 1.6 fold-increase in uptake compared to control (Fig. 3a, lower panel). Because such substrates are not exclusive to their efflux pumps and may interact with other efflux pumps, we further confirmed the presence of efflux pump activity using doxorubicin, a substrate for all three pumps (Fig. 3b). With the exception of a twofold increase in doxorubicin net uptake following CsA treatment in the T-Matrigel group, we did not notice any significant differences in the net uptake between the different Matrigel groups. We noted an increase of 1.3 to 1.4-fold compared to control, which is consistent with previous literature [7]. In conclusion, the source of Matrigel influenced the barrier outcome in IMR90-derived BMECs in terms of barrier tightness but not in terms of drug efflux-mediated transport.

### Experiments using essential-8 medium

**Fig. 4:**
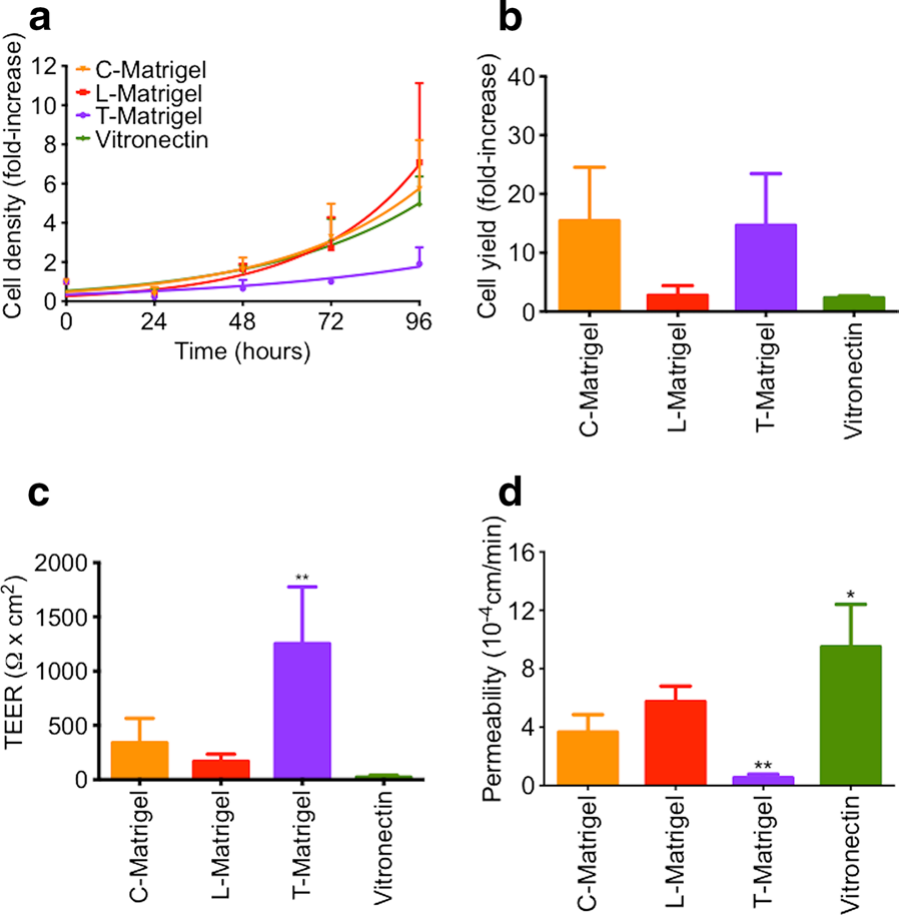
Essential-8 medium does not impact BMECs differentiation. **a** IMR90 iPSC growth curve on C-Matrigel (*orange*), L-Matrigel (*red*), T-Matrigel (*purple*) or vitronectin (*green*)-coated surfaces. IMR90 iPSCs were seeded as single cells at day 0 at a density of 20 × 10^3^ cells/cm^2^ on the different types of matrices and maintained in E-8 medium. At each timepoint (day), IMR90 iPSC colonies were dissociated as single cells using Accutase and counted with a cell hemocytometer using 0.4 % Trypan blue as an exclusion dye. **b** Cell yield at day 8 of differentiation. Cell yield was determined by dividing average density at day 8 of differentiation by the initial cell seeding density at day 0. **c**, **d** TEER and sodium fluorescein permeability values on purified iPSC-derived BMECs monolayers 48 h after purification. *Note* the poor barrier properties in cells differentiated on Geltrex (L-Matrigel) or on vitronectin-N (vitronectin), * and ** denote *P* < 0.05 and *P* < 0.01 in comparison to C-Matrigel

**Fig. 5:**
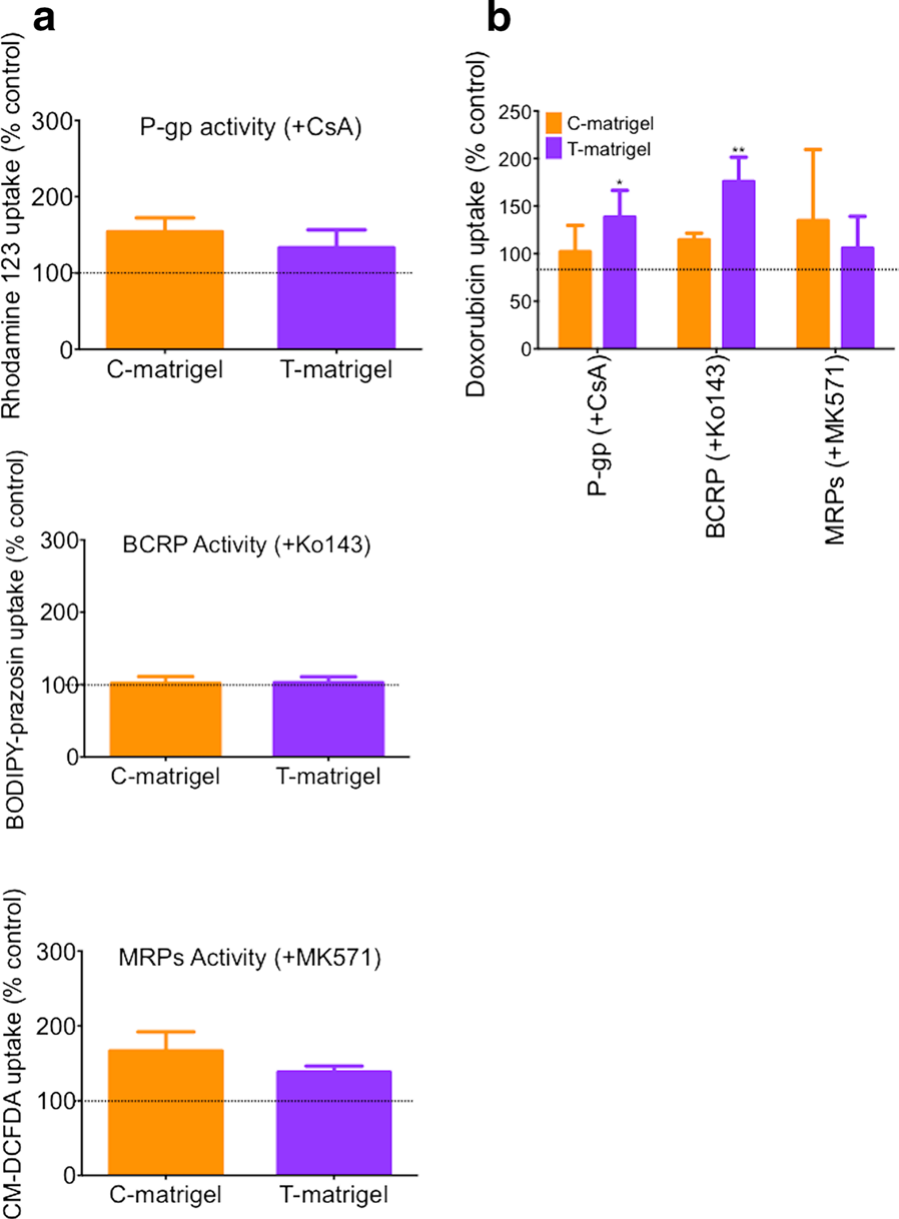
Essential-8 medium. **a** Drug uptake profile of rhodamine 123, BODIPY-prazosin and CM-DCFDA in presence of CsA, Ko143 or MK571. Drug uptakes of the same efflux substrates in absence of inhibitors were used as controls. Fluorescence in controls was arbitrarily set to 100 %. **b** Doxorubicin drug uptake profile in presence of CsA, Ko143 or MK571, * and ** denote *P* < 0.05 and *P* < 0.01 in comparison to C-Matrigel

Because GFR-Matrigel from different sources displayed variable outcomes on IMR90 differentiation into BMECs, and also that the production of B-Matrigel has been discontinued, we investigated alternative culturing conditions that can circumvent these issues. In particular, we investigated the possibility to change the BMEC differentiation protocol using defined conditions with, E-8 medium and two additional ECMs, vitronectin and L-Matrigel (Fig. 4). Undifferentiated IMR90 iPSCs grown on C-Matrigel in presence of E-8 medium (Fig. 4a, orange line), showed a doubling time similar to mTeSR (26.72 and 27.28 h respectively), as well as cells grown on Geltrex [L-Matrigel (red line), 20.31 h] and Vitronectin-N [Vitronectin (green line), 29.69 h]. However, cells grown on T-Matrigel (purple line) in combination with E-8 medium displayed a much reduced growth rate compared to mTeSR with an average doubling time of 41.05 h. By maintaining the same seeding density as previously mentioned, we obtained after 8 days of differentiation an average BMEC yield (Fig. 4b) of 15.45 ± 9.08 and 14.66 ± 8.78-fold increase in cells grown in C-Matrigel and T-Matrigel respectively. Surprisingly, cells grown on L-Matrigel or on Vitronectin were incapable of reaching a yield that was higher than twofold. Next we compared the average barrier function of these monolayers by measuring changes in TEER and permeability (Fig. 4c, d). Maintenance of IMR90 iPSCs in E-8 did not interfere with the BMEC barrier tightness, as we noted TEER values (305.4 ± 85.09 and 1254 ± 523.7 Ω cm^2^) and permeability (3.66 ± 1.20 and 0.54 ± 0.13 × 10^−4^ cm/min) for C and T-Matrigels, similar to those reported in IMR90 iPSCs maintained in mTeSR media. However, cells differentiated on L-Matrigel and Vitronectin showed poor barrier properties, as noted by lower TEER (169.0 ± 32.86 and 23.75 ± 16.99 Ω cm^2^) and higher permeabilities (5.76 ± 1.05 and 9.50 ± 2.90 × 10^−4^ cm/min). Finally, we investigated changes in drug efflux activity by measuring changes in rhodamine 123, FL-BODIPY prazosin and CM-DCFDA uptake in cells grown on both C- and T-Matrigel (Fig. 5). We observed similar trends to cells maintained in mTeSR prior to differentiation (Fig. 5a), although the net uptake values for each pumps were slightly lower than IMR90 iPSCs maintained in mTeSR. Such decreased drug efflux pump activity was also observed in the net uptake of doxorubicin (Fig. 5b), especially in cells differentiated on C-Matrigel. In conclusion, our data suggest that the nature of the medium used in maintenance of pluripotent stem cells has little impact on the differentiation process. However, the replacement of Matrigel by a defined ECM has a detrimental effect on BMEC differentiation.

## Discussion

In the last few years, in vitro models based on patient-derived iPSCs have gained a sizable momentum in modeling neurodegenerative disorders and certain types of epilepsies [22–25]. More recently, the publication of stem cell-based models of the human BBB brought a complementary approach to other in vitro models based on human primary cultures or on the hCMEC/D3 immortalized cell line [7–9, 11, 26–29]. Such patient-specific and disease-specific sources of cells may provide a valuable tool in modeling the impact of genetic disorders at the BBB and lead to a better understanding of how such disorders may result in the dysfunction of one or several components of the neurovascular unit (e.g. astrocytes, neurons, BMECs).

In their previous studies, Shusta and colleagues have established the method and demonstrated the ability to obtain BMECs from both embryonic and iPS stem cell lines with variable outcomes [7, 8, 11]. They used GFR-Matrigel and mTeSR as a foundation for the differentiation. In this study, we investigated the impact of GFR-Matrigel from different sources and xeno-free culturing conditions (E-8 medium and Vitronectin) on BMEC differentiation using IMR90-c4, a human iPSC line [13]. In particular, the main driving force for our study was primed by the discontinuation of B-Matrigel production and its replacement by C-Matrigel.

Although the composition of GFR-Matrigel suggests a reduction in ECM-bound growth factors, a recent comparative proteomic study conducted by Hughes and colleagues between conventional and GFR-Matrigel has highlighted notable differences in their chemical composition [15]. Interestingly, the authors identified over 400 peptides that were exclusively found inside the GFR-Matrigel and they also found signatures of proteins found naturally in cytoplasmic or nuclear compartments. Based on this study and our data, we can speculate that Matrigel composition may make an important contribution to BMEC differentiation. We speculate that cell-ECM interactions with integrins maybe an important driving force in this differentiation process, as we were not able to obtain any satisfactory differentiation when cells were grown on vitronectin-N. However, we cannot exclude the presence of non-ECM factors (e.g. growth factors) retained by the GFR-Matrigel that may influence such differentiation.

An interesting feature observed in our study was the notable decrease in barrier tightness when cells were grown on C-Matrigel. On this ECM, differentiating iPSC colonies failed to form neural tracts as observed by Shusta and colleagues [7, 8, 11]. Indeed, IMR90 differentiating colonies on this substrate showed a macroscopical profile similar to the low-density (10 × 10^3^ cells/cm^2^) group described by Wilson et al. [11], although we initiated our differentiation at a much higher cell density (100 × 10^3^ cells/cm^2^). Barrier properties from BMEC monolayers differentiated in C-Matrigel shared similar values to BMECs purified from the low-density group, as marked by low TEER values (~300 Ω cm^2^). Notably such values coincide with those reported in the seminal study by Lippmann et al. [8], in which BMEC differentiation was performed in absence of retinoic acid (RA). RA has been documented as a barrier inducer in hCMEC/D3 cell monolayers [30], yet the induction range was much more modest compared to IMR90-derived BMECs. We speculate that in our model, neuron progenitors described in such neural tracts might be secreting RA-induced factors resulting in the barrier tightening.

Finally, we observed significant differences between matrices in terms of undifferentiated stem cell doubling time thus suggesting differences in growth factors intrinsic to GFR-Matrigel sources. GFR-Matrigel differs from regular Matrigel by an additional treatment with ammonium sulfate. Such treatment results in the reduction of growth factors bound to the extracellular matrix components contained within the Matrigel [31]. Thus, we speculate that one source of variability may originate from this essential step in the manufacturing of the GFR-Matrigel. In this study, among the three different manufacturers providing stem cell qualified Matrigel, only one manufacturer (Trevigen) provided Matrigel that enabled IMR90-derived BMEC monocultures to have barrier properties similar to the existing literature.

## Conclusion

This study confirms the ability to differentiate iPSC-derived BMECs from the IMR90-c4 cells as previously reported. Whereas the effect of stem cell maintenance medium has little impact on BMEC differentiation, variations in the production and manufacturing of GFR-Matrigel raised important issues that should foster further research. A transition from empirically-formulated ECMs into a highly-defined synthetic scaffold would ensure consistent BMEC differentiation using a xeno-free, growth factor-independent medium.
